# Native and Alien Antarctic Grasses as a Habitat for Fungi

**DOI:** 10.3390/ijms25158475

**Published:** 2024-08-03

**Authors:** Sebastian Piłsyk, Urszula Perlińska-Lenart, Anna Janik, Patrycja Skalmowska, Anna Znój, Jan Gawor, Jakub Grzesiak, Joanna S. Kruszewska

**Affiliations:** 1 Institute of Biochemistry and Biophysics, Polish Academy of Sciences, Pawińskiego 5A, 02-106 Warsaw, Poland; seba@ibb.waw.pl (S.P.); lenart@ibb.waw.pl (U.P.-L.); annaj@ibb.waw.pl (A.J.); eszeweria@vp.pl (P.S.); aznoj@ibb.waw.pl (A.Z.); gaworj@ibb.waw.pl (J.G.); 2 Botanical Garden—Center for Biological Diversity Conservation, Polish Academy of Sciences, Prawdziwka 2, 02-973 Warsaw, Poland

**Keywords:** *Deschampsia antarctica*, *Poa annua*, Antarctic fungi, hydrolytic activity

## Abstract

Biological invasions are now seen as one of the main threats to the Antarctic ecosystem. An example of such an invasion is the recent colonization of the H. Arctowski Polish Antarctic Station area by the non-native grass *Poa annua*. This site was previously occupied only by native plants like the Antarctic hair grass *Deschampsia antarctica*. To adapt successfully to new conditions, plants interact with soil microorganisms, including fungi. The aim of this study was to determine how the newly introduced grass *P. annua* established an interaction with fungi compared to resident grass *D. antarctica*. We found that fungal diversity in *D. antarctica* roots was significantly higher compared with *P. annua* roots. *D*. *antarctica* managed a biodiverse microbiome because of its ability to recruit fungal biocontrol agents from the soil, thus maintaining a beneficial nature of the endophyte community. *P*. *annua* relied on a set of specific fungal taxa, which likely modulated its cold response, increasing its competitiveness in Antarctic conditions. Cultivated endophytic fungi displayed strong chitinolysis, pointing towards their role as phytopathogenic fungi, nematode, and insect antagonists. This is the first study to compare the root mycobiomes of both grass species by direct culture-independent techniques as well as culture-based methods.

## 1. Introduction

Fungi form widespread symbiotic associations with plants. Their colonization of plants is promoted by the limited availability of phosphorus and/or nitrogen, as fungi play a major role in plant nutrition, growth, water absorption, nutrient cycling, and protection against pathogens [1]. Thanks to mycorrhizal fungi, colonized plants gain access to a wide range of nutrients such as phosphorus, nitrogen, and microelements (S, K, Ca, Fe, Cu, Zn) from a larger source area via roots expanded by branched fungal hyphae [2,3]. Additionally, the extraradical mycelium absorbs mineral nutrients from the soil and transports them to the host plant. Thus, fungi mitigate the effects of abiotic stresses such as drought, nutrient imbalance, and unfavorable temperature on plant growth [4]. Plants actively recruit fungi from their environment using strigolactones released by roots to induce fungal spore germination and hyphal growth towards the host roots [5]. 

Endophytic fungi colonize plant tissues internally and have the potential to act as control agents, such as biological agents or elicitors in the process of induced resistance and in attenuating abiotic stresses [3,6]. Endophytic fungi inhabit a similar ecological niche to that occupied by phytopathogens, thus being able to protect their environment and control them through competition, production of antagonistic substances, direct parasitism, and even inducing resistance or tolerance in plants [6].

Fungi also benefit from symbiosis with plants by taking nutrients, carbohydrates, and lipids from their hosts [1,7,8,9]. Up to 20% of photoassimilates in host plants flow to mycorrhizal root systems to support the mutualistic interaction between hosts and fungi [1]. Evidence suggests that the fungus receives carbon from the plant in the form of sugars (hexoses); however, plants could transfer alternative carbon sources such as lipids [1,7,8,9]. In addition, Helber et al. [8] supported the hypothesis that the exchange of carbon for phosphate between plants and fungi is tightly linked. 

Fungi are believed to be able to colonize a large number of host plant species, indicating a lack of host specificity [7]. Different combinations of host plants and fungi have different effects on various aspects of symbiosis, suggesting a high functional diversity of mycorrhizal fungi [10]. Different community compositions of fungi affect plants differently and play a major role in ecosystem biodiversity, sustainability, and productivity. 

Traditionally, fungi are viewed as plain decomposers of complex organic compounds in the environment. However, mycorrhizal fungi should be redefined from decomposers to producers or assistant producers of plants, increasing the productivity and fitness of plants in ecosystems. The protective properties of mycorrhizal fungi are particularly important in the adaptation of plants to the extremely difficult environment of Antarctica [4]. The soils of the H. Arctowski Polish Antarctic Station are characterized by low nutrient availability, imposing an additional stress that plants must deal with in order to survive [11]. These extreme conditions also influence fungi. They can function in the coldest environment owing to their great efficiency of adaptation and combination of strategies such as the production of cold-active enzymes, unsaturated lipids, melanin, antifreeze proteins, and the cryoprotectants glycerol, trehalose, and sugar alcohols (mannitol, arabitol, erythritol) [12]. 

Only two species of grasses are currently found in Antarctica, i.e., *Deschampsia antarctica* Desv. (Antarctic hair grass) and *Poa annua* L. (annual bluegrass), both belonging to the family *Poaceae*. 

The global occurrence of *D. antarctica* is very limited. It has been found in South America, Orkney, South Shetland, and along the western Antarctic Peninsula, while *P. annua*, an opportunistic species, has spread worldwide. The species was initially recorded in the vicinity of the Polish Antarctic Station in the austral summer of 1985/86 [13]. Amplified fragment length polymorphism (AFLP) analysis showed that the Antarctic population of *P. annua* was a subpopulation of the Polish one [14,15].

Both grasses have adapted to the extreme Antarctic conditions by symbiosis with bacteria and fungi [3,4,11]. Znój et al. [11] draw attention to *Flavobacteriaceae* suspected to be major bacterial contributors to the ecological success of *P. annua*, especially in harsh Antarctic conditions. Some authors point out that endophyte-infected grasses are more competitive than non-infected grasses and thrive better than non-infected grasses with limited resources [3,4]. 

In this study, we investigated root-associated fungi from *D. antarctica* and *P. annua* collected in the vicinity of the H. Arctowski Polish Antarctic Station, located at the western shore of Admiralty Bay, King George Island (South Shetlands Islands, maritime Antarctica). The aim of this study was to compare the communities of fungi inhabiting the roots of two different grasses and to learn how the fungal microbiome of a grass adapting to new environmental conditions is shaped. Our working hypothesis states that the composition of the fungal endorhizome communities of both investigated grass species differs considerably from those associated with *D*. *antarctica* roots, displaying a more uniform structure, while the structure of those associated with *P*. *annua* roots differs depending on the sampling site.

## 2. Results

### 2.1. Fungal Diversity in Antarctic Grasses and the Rhizosphere Soil

To characterize the fungal microbiome associated with roots of *D. antarctica* and *P. annua*, we analyzed the ITS1 (internal transcribed spacer) region of rDNA in total DNA obtained from samples of grass roots and surrounding soil collected in the Ecology Glacier foreland and at two locations in the Arctowski Station area. 

**Figure 1:**
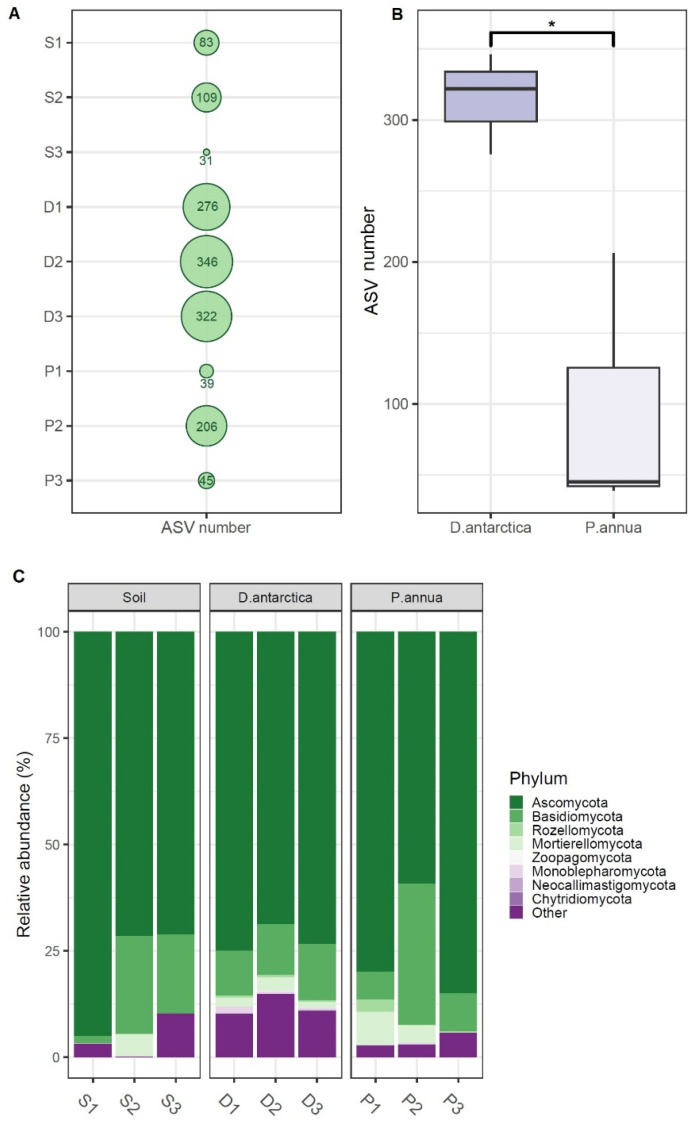
Fungal phylogenetic diversity in *P. annua* roots, *D. antarctica* roots, and the associated soil: (**A**) average ASV numbers per site; (**B**) differences in ASV numbers between *P. annua* and *D. antarctica* roots; and (**C**) relative abundance of fungal sequences identified to the phylum level. S1–S3—soil samples; D1–D3—*D. antarctica* root samples; P1–P3—*P. annua* root samples; *—significant at *p* < 0.05.

Obtained ASV (Amplicon Sequence Variant) numbers varied between 31 and 346. In the soil samples, they equated to an av. of 74.3 ± 39.7, in *D. antarctica* roots, an av. of 314.6 ± 35.6, and in *P. annua* roots, an av. of 96.7 ± 94.7. Sites 2 and 3 displayed the highest and lowest fungal phylogenetic diversity, respectively (Figure 1A). ASV numbers in *D. antarctica* roots were significantly higher than those found in *P. annua* roots at *p* < 0.05 (Figure 1B). Ascomycota dominated all the samples in terms of sequence contribution (av. 75.4 ± 10.3%) with Basidiomycota as the second most numerous phylum (av. 14.2 ± 9.5%). Ascomycota’s relative abundance fluctuated considerably in the soil samples (av. 79.2 ± 13.7%) and *P. annua* roots (av. 74.7 ± 13.6%) when compared with *D. antarctica* roots (av. 72.4 ± 3.2%). Basidiomycota’s contribution displayed a similar pattern as follows: soil—av. 14.4 ± 11.3%, *P. annua* roots—av. 16.2 ± 14.7%, and *D. antarctica*—av. 11.9 ± 1.3%. Mortierellomycota maintained a stable persistence only in *D. antarctica* roots (av. 2.3 ± 0.9%) while displaying varying contributions to soil (av. 1.8 ± 3.1%) and *P. annua* root (av. 3.9 ± 3.8%) communities. The phylum Rozellomycota was present in roots in relatively small amounts (av. 0.8 ± 0.9%) while being almost absent in the soil samples (av. 0.003 ± 0.005%) (Figure 1C).

**Figure 2:**
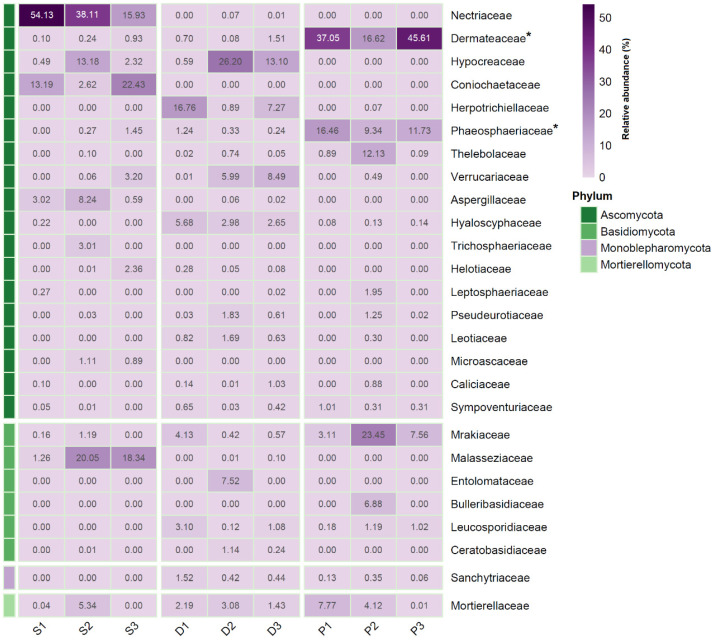
Relative abundance of fungal sequences identified to the family level. *—families that significantly (*p* < 0.05) differed in abundance between *P. annua* and *D. antarctica*/soil samples; S1–S3—soil samples; D1–D3—*D. antarctica* root samples; P1–P3—*P. annua* root samples.

The soil samples were most abundant in the Ascomycota families of Nectriaceae (av. 36.1 ± 19.2%) and Coniochaetaceae (av. 12.7 ± 9.9%), with the Basidiomycota family of Malasseziaceae (av. 13.2 ± 10.4%) also displaying a considerable contribution. Several Ascomycota families displayed a high contribution on average to *D. antarctica* root fungal communities, albeit their abundance differed considerably among sampling sites as follows: Hypocreaceae—av. 13.3 ± 12.8%, Herpetrichiellaceae—av. 8.3 ± 7.9%, and Verrucariaceae—av. 4.8 ± 4.3%. However, the sequence contribution of the family Mortierellaceae displayed a somewhat even distribution in *D. antarctica* roots (av. 2.2 ± 0.8%). The most abundant fungal families within *P. annua* roots were Dermataceae (av. 33.1 ± 14.9%) and Phaeospaeriaceae (av. 12.5 ± 3.6%). The relative abundances of those two families were significantly higher (*p* < 0.05) in *P. annua* roots than in the roots of *D. antarctica* and in the examined soil samples. *P. annua* roots also harbored considerable amounts of the Basidiomycota family Mrakiaceae (av. 11.4 ± 10.7%) (Figure 2).

**Figure 3:**
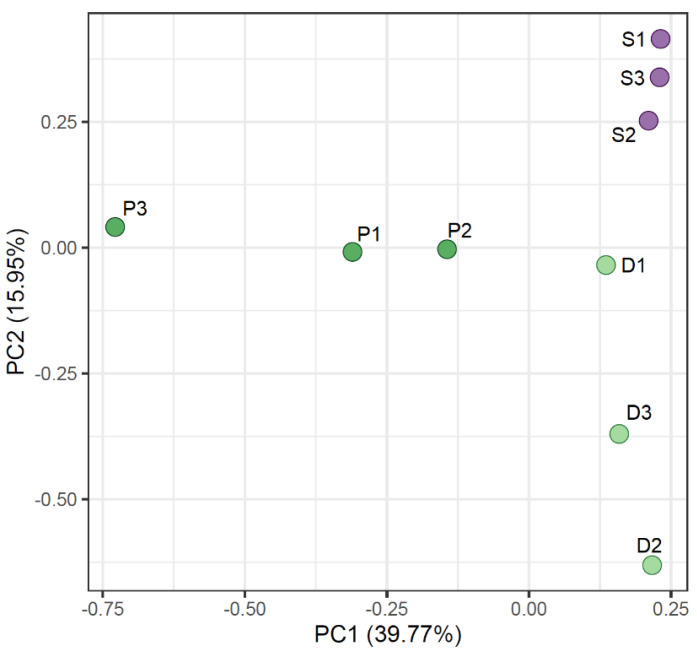
Principal Component Analysis (PCA) based on relative ASV abundances of obtained fungal sequences. S1–S3—soil samples; D1–D3—*D. antarctica* root samples; P1–P3—*P. annua* root samples.

The Principal Component Analysis (PCA) clustered the samples based on ASV abundances (Figure 3). The soil samples are clustered tightly in the right upper corner of the graph. The root samples showed a less organized placement on the PCA, showcasing a high degree of variation not only among grass species but also among sampling sites.

**Figure 4:**
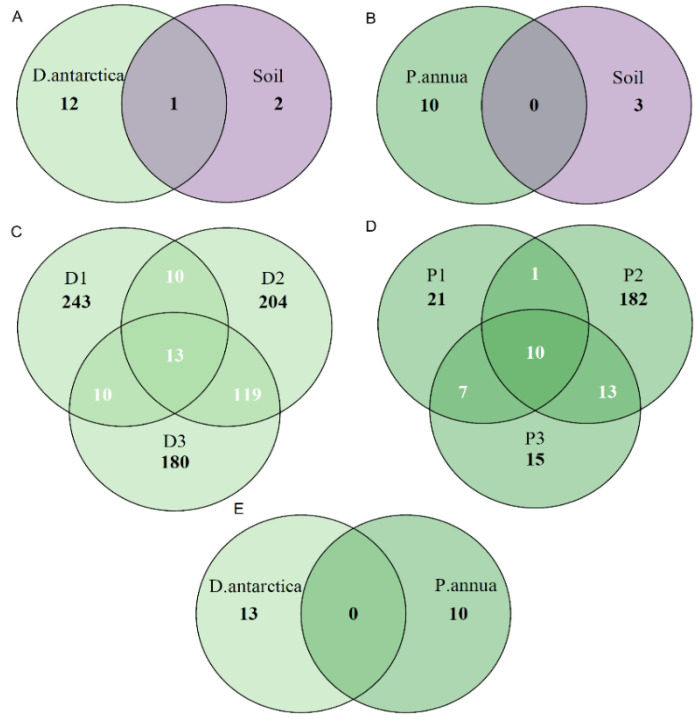
Venn diagrams displaying shared ASVs. (**A**) ASVs shared between core fungal communities of *D. antarctica* and the associated soil communities; (**B**) ASVs shared between core fungal communities of *P. annua* and the associated soil communities; (**C**) ASVs shared within the fungal communities of *D. antarctica* from different sites; (**D**) ASVs shared within the fungal communities of *P. annua* from different sites; and (**E**) ASVs shared between the core communities of *D. antarctica* and *P. annua*. S1–S3—soil samples; D1–D3—*D. antarctica* root samples; P1–P3—*P. annua* root samples. The core microbiome was defined as a set of specific ASVs that occurred in every sample within a particular group.

The Venn diagrams presented in Figure 4 show the amount of ASVs shared among the samples and sample sets. While *D*. *antarctica* shared 1 ASV between its root community and the examined Antarctic soil community (Figure 4A), *P*. *annua* shared none (Figure 4B). The fungal core microbiome of *D*. *antarctica* roots consisted of 13 ASVs (Figure 4C), while in the case of *P*. *annua*, the core microbiome comprised 10 ASVs (Figure 4D). No ASVs were shared between core microbiomes of the two grass species (Figure 4E). 

**Figure 5:**
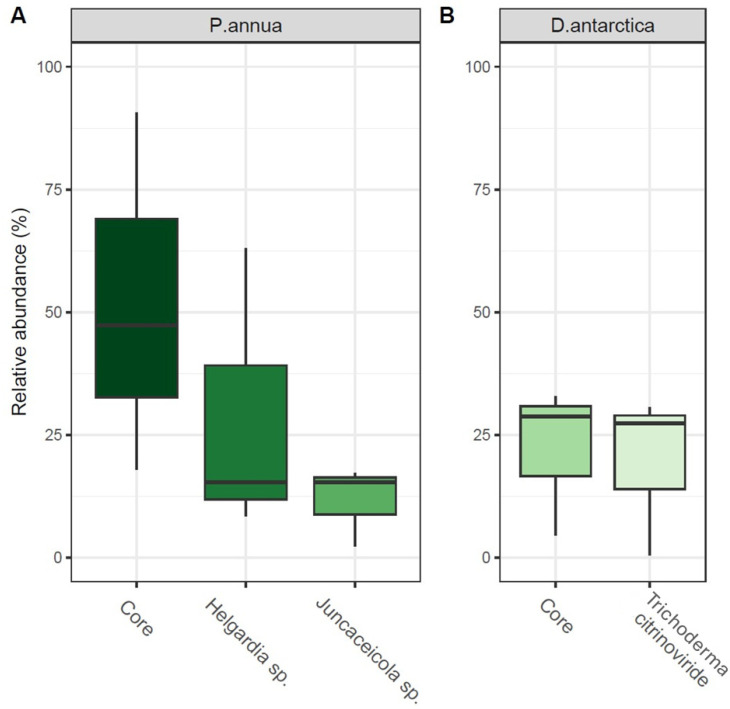
Size of fungal core communities and their major contributors in (**A**) *P. annua* roots and (**B**) *D. antarctica* roots. The size of the fungal core microbiome (core) is displayed as the relative sequence abundance (%) of 10 shared ASVs within *P. annua* roots and 13 shared ASVs within *D. antarctica* roots. Major contributors to the core microbiomes are displayed as relative sequence abundances of particular ASVs that made a substantial contribution to their respective core communities. Phylogenetic affiliations of those ASVs are displayed as identified by the Unite v.9.0 database.

The fungal core microbiome of *P*. *annua* roots (10 ASVs) comprised between 18 and 90% of the community’s relative abundance. Its most numerous members were identified as *Helgardia* sp. (f. *Dermataceae*)—8.5 to 63% and *Juncaceicola* sp. (f. *Phaeospaeriaceae*)—2.3 to 15.3% (Figure 5A). The core microbiome of *D*. *antarctica* roots (13 ASVs) comprised between 4.5 and 32.9% of the total fungal community. The most abundant ASV (0.5–30.6% of total community) was identified as the species *Trichoderma citrinoviride* (f. *Hypocreaceae*), which represents the one ASV shared between *D*. *antarctica* roots and the Antarctic soil (Figure 5B). 

### 2.2. Fungi Identified to the Species Level from Grass Roots and Their Characteristics

Forty-two and eighty-nine fungal sequences associated with *P. annua* and *D. antarctica* roots, respectively, were identified to the species level. Overall, 65.1% of the identified fungi belonged to the phylum Ascomycota, 26.4% to Basidiomycota, and 8.6% to Mortierellomycota.

**Figure 6:**
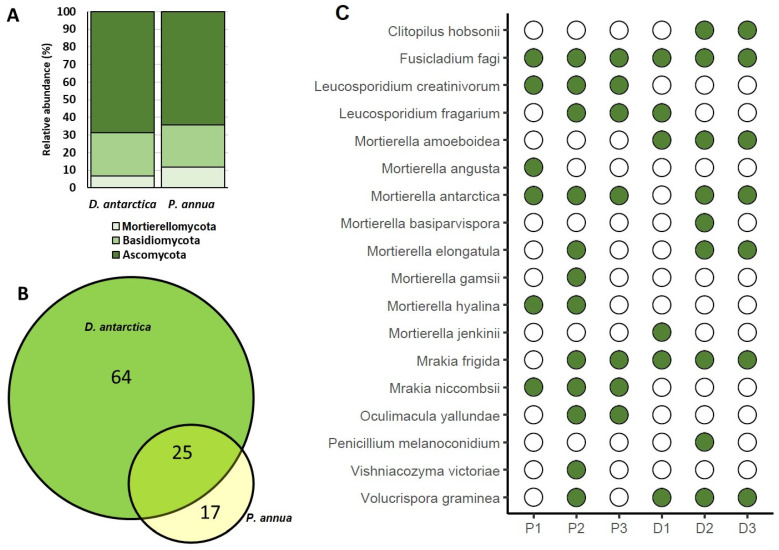
Fungal species identified in *P. annua* and *D. antarctica* roots: (**A**) phylum-rank affiliation of identified species (as % contribution within the group); (**B**) number of individual and shared identified species; and (**C**) presence (green circles)–absence (white circles) matrix of selected fungal species in *P. annua* (P1–P3) and *D. antarctica* (D1–D3) roots. The full list of identified strains is provided in Table S1.

Twenty-five species were recruited by both grasses. Among the most abundant genus *Mortierella* (Mortierellomycota), represented by nine species, *M. amoeboidea*, *M. jenkinii*, *M. minutissima*, and *M. basiparvispora* were identified in *D. antarctica*, and *M. angusta*, *M. hyalina* and *M. gamsii* were associated with *P. annua* roots. Both grasses recruited *M. antarctica* and *M. elongatula*. *Mortierella* was the only identified genus belonging to the Mortierellomycota phylum. Although many more species were identified in the roots of *D. antarctica* compared with *P. annua*, the relative abundance of phylum members in the roots of both grasses was almost the same (Figure 6, Table S1).

### 2.3. Cultivable Fungi Inhabiting Grasses in Antarctica and Their Hydrolytic Activity

To study the enzymatic activities of the Antarctic fungi experimentally, they were cultured directly from the grass and soil samples from all the locations. Few fungi from the roots of *D. antarctica* or *P. annua* from Arctowski Station 2 could be cultivated, while all of those from the soil came from Arctowski Station 2 and the Ecology Glacier foreland (Table 1 and Table S2). 

Most of the cultivable fungi belonged to Ascomycota, and only four and three species to Basidiomycota and Mortierellomycota, respectively. 

To survive in the Antarctic, soil fungi have to be able to utilize complex carbon sources, which they achieve by producing polymer-degrading enzymes. To analyze the hydrolytic potential of cultivable fungi, we determined their ability to grow on plant-derived polysaccharides such as cellulose, xylan, starch, and pectin, which can be found in the soil and on chitin potentially derived from the cell wall of fungi or arthropods exoskeleton [35,36,37].

**Figure 7:**
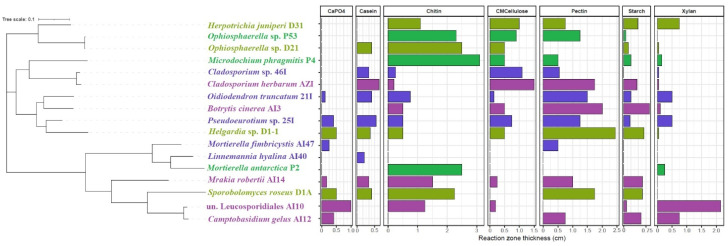
Taxonomic affiliation of fungal strains isolated from Antarctic soil and grass roots with annotated metabolic features. CaPO_4_—tricalcium phosphate; CMCellulose—carboxymethylcellulose; green—*P*. *annua* strains; yellow-green—*D*. *antarctica* strains; purple—soil strains (Ecology Glacier); violet—soil strains (Arctowski Station).

The availability of nutrients in cold ecosystems depends strongly on the soil temperature and moisture and the activity of soil microbiota, which in turn control nitrogen fixation, mineralization, and organic matter decomposition [38,39,40,41,42]. Plant growth can be limited by nitrogen availability. The ability of fungi to secrete proteases and make the inaccessible organic nitrogen pool available to plants was analyzed. We determined the proteolytic potential of the cultivable grass root and soil fungi (Figure 7). 

Phosphorus is often the limiting nutrient present in the soil in an insoluble form inaccessible to plants. Notably, some fungi secrete organic acids that mobilize the insoluble phosphates. We determined the ability of some of the grass-associated and soil fungi to convert insoluble phosphorite (Ca_3_(PO_4_)_2_) to an absorbable form (Figure 7). 

Seventeen fungal strains isolated from the examined samples were identified phylogenetically and subjected to agar plate-based evaluation of seven metabolic features (Figure 7). Seven strains were isolated from grass roots, while ten were isolated from the Antarctic soil. All root-derived strains were capable of chitin digestion, with *Microdochium phragmitis* P4 being the most potent chitinolytic strain. Seven soil-derived strains also displayed this ability albeit to a lesser extent, with *Mrakia robertii* AI14 showing the greatest chitin dissolution activity within this group. Five root and seven soil strains produced cellulases, most notably, *Herpotrichia juniperi* D31 (roots) and *Cladosporium herbarum* AZI (soil). Pectin was digested by five root strains and eight soil strains with the highest scores noted for *Helgardia* sp. D1-1 and *Botrytis cinerea* AI3, respectively, for root and soil isolates. Starch was digested by six root strains and seven soil strains. *Helgardia* sp. D1-1 and *Botrytis cinerea* AI3 were the most potent amylolytic strains. Among the five root strains that degraded xylan, *Herpotrichia juniperi* D31 was the most active, while among the seven soil xylan-degrading strains, isolate AI10 (un. *Leucosporidiales*) was the most potent. Tricalcium phosphate was solubilized by only two root-derived isolates, including *Helgardia* sp. D1-1 and *Sporobolomyces roseus* D1A, while seven soil strains displayed this ability, with strain AI10 displaying the highest activity. Casein was enzymatically degraded by three root isolates and six soil isolates, where *Ophiosphaerella* sp. D21 and *Sporobolomyces roseus* D1A were the most active among root strains and *Cladosporium herbarum* AZI was the most active among soil strains. Two soil strains (*Oidiodendron truncatum* 21I and *Pseudoeurotium* sp. 25I) and one root strain (*Helgardia* sp. D1-1) displayed all seven of the examined traits. 

## 3. Discussion 

*Deschampsia antarctica* has been present in the Antarctic region since at least the late Pliocene (≈3.6 Mya) [43]. In this study, the reported high phylogenetic diversity in *D*. *antarctica* roots (based on ASV numbers) is likely explained by the long history of interaction between it and Antarctic soil microflora [44]. The relatively stable proportions of phylum-rank sequences in its roots also corroborate the notion of an established microbiome [45]. However, PCA clustering suggests a dynamic reorganization of the fungal endophyte community depending on the sampling site. Despite significant discrepancies in the phylogenetic structure, a core fungal microbiome was recognized, composed largely of one ASV, which was identified as *Trichoderma citrinoviride*. Interestingly, the relative abundance of the core microbiome taxa within *D*. *antarctica* roots was as low as 4.5%, suggesting that those endophytes are not an indispensable part of the community, i.e., not pivotal for the growth and development of the host plant. In this regard, Upson et al. [46] showed large variations in fungal endophyte colonization in *D*. *antarctica* roots procured from the South Shetland Islands. Furthermore, there was a continued presence of this *T*. *citrinoviride* ASV in the examined Antarctic soil samples, meaning that the fungus was not exclusively housed in hair grass roots, with soil being its primary and most accessible reservoir. The role of *T*. *citrinoviride* in the tissues of *D*. *antarctica* remains elusive, as this is the first record of its presence in this plant species and even in Antarctica. However, what is known about *T*. *citrinoviride* and, in general, about the *Trichoderma* genus is their ability to parasitize other fungi, most notably, severe plant pathogens, acting as a biocontrol agent and, consequently, promoting plant health [47]. The relative abundance of the *T*. *citrinoviride* ASV displayed a positive correlation with the fungal phylogenetic diversity (ASV numbers) in *D*. *antarctica* roots, implying that no antagonistic processes were engaged. However, a study from 2018 showed that pathogen-stressed tomato and cucumber plants promoted the growth of *Trichoderma harzianum*, while their root exudates induced positive chemotropic responses in germinating *T*. *harzianum* spores [48]. This could be a plausible scenario for *D*. *antarctica* roots, considering that the relative abundance of Hypocreaceae (*Trichoderma* parent taxon) displayed positive correlations with several other fungal families residing in *D*. *antarctica* roots, most notably, the family Pseudeurotiaceae, which largely consisted of sequences identified as *Pseudogymnoascus destructans* in this study. Although this species of fungus is the causative agent of the bat disease called white-nose syndrome, a study by Meteyer et al. [49] concluded that the ancestral state of the clade containing *P*. *destructans* was a plant-associated fungus with invasion strategies compatible with ancestral contributions from plant pathogens. Interestingly, endophyte-free specimens of *D*. *antarctica* displayed a higher Relative Competition Index when grown with *P*. *annua* than those infected with native fungal endophytes, albeit this phenomenon was only observed for the ones sampled from maritime Antarctica [50]. Presumably, *D*. *antarctica,* because of its evolutionary adaptations to Antarctic conditions, does not have to depend on any specific set of fungal endophytes to survive, yet it tolerates a large number of fungal species in its roots, promoting inter-species competition to maintain homeostasis. Similarly, Znój et al. [51] found a biodiverse bacterial community within the boundaries of the *D*. *antarctica* root system, with the root endosphere colonized by the known plant pathogen *Clavibacter michiganensis* and also by substantial amounts of bacterial *Polyangiaceae* family members, most of which are documented to exhibit a predatory lifestyle and secrete antimicrobial secondary metabolites [52]. 

*P*. *annua* is not native to Antarctica [53]. Its first sightings in the H. Arctowski station area were made during the austral summer of 1985–86 [54]. Soils imported for greenhouse cultivation were the likely source of its introduction. Since then, *P*. *annua* has established its presence in the general area, with new genetic material being incorporated into the population because of ongoing human activity [15]. Despite the systematic eradication of this species, it persists in the region [55]. In this study the fungal community in the roots of *P*. *annua* displayed site-specific variations both in phylogenetic diversity as well as structure, suggesting a relatively labile community, largely dependent on the present edaphic conditions. However, a core microbiome was distinguishable within the examined *P*. *annua* roots, albeit its relative abundance varied considerably (18–90%). Interestingly, the core taxa contribution negatively correlated with the general fungal diversity, hinting towards a possible antagonistic interaction. The two major contributors to the *P*. *annua* core community (two ASVs identified as *Helgardia* sp. and *Juncaceicola* sp.) were not present in the core communities of *D*. *antarctica* roots nor the Antarctic soil samples, suggesting specific affinity towards tissues of the annual bluegrass. A study by Molina-Montenegro et al. [50] on fungal involvement in the invasiveness of *P*. *annua* in Antarctica clearly showed a significant (*p* < 0.0001) positive influence of native endosymbionts on seed germination, survival, and flower production. As the potting soil in this experiment was sterile, the endosymbionts presumably aided the grass mainly in coping with abiotic environmental stressors by modulating plant gene expression and triggering the plant stress response. Consequently, beneficial plant–microbial symbioses should increase in prevalence in more stressful environments. Indeed, the contribution of *Helgardia* sp. and *Juncaceicola* sp. to the *P*. *annua* root microbiome was the highest in site 3, which was located at the forefield of Ecology Glacier, characterized by limited organic matter as well as frequent freeze–thaw and wet–dry cycles [56]. This is similar to the findings of Znój et al. [11], where the *P*. *annua* root endospheric bacteriome of Ecology Glacier forefield specimens was dominated by only three bacterial families, most notably, *Flavobacteriaceae*. Members of this family are known to produce ACC deaminase, which lowers the concentration of the plant stress hormone ethylene, a substance that inflicts a significant reduction in plant growth and development, especially in cold-stressed plants [57,58]. Representatives of the genera *Helgardia* and *Juncaceicola* were isolated from Antarctic materials, including soils and *D*. *antarctica* leaves and roots [59,60]; however, their role as endophytes has not been elucidated. Furthermore, it is not clear if these two core fungal ASVs in question are native to the Antarctic environment or if they were embedded in the original seeds that started the examined *P*. *annua* population. Further research is needed on this matter; however, it is apparent that *P*. *annua* has a different strategy for managing its fungal endophyte community than the Antarctic native *D*. *antarctica*. 

Some fungi were identified to the genus/species level in the microbiome analysis, and some of them were also identified among cultivable fungi. The Mortierellaceae family was represented in the roots of both grasses, and most of the fungi from this family identified to the species level belong to the *Mortierella* genus (Supplementary Table S2). *Mortierella* spp. are widespread in Antarctica and have been found in lake waters, soil, glaciers, plants, algae, lichens, and mosses [20,21]. They are acid-tolerant producers of fatty acids and other organic acids needed to solubilize rock phosphate [22]. This ability allows *Mortierella* to grow in environments deficient in soluble inorganic phosphorus and makes it important in sediment decomposition and nutrient recycling [23]. Phosphate-solubilizing microorganisms convert insoluble phosphates to a soluble form available for absorption and use by other organisms, mainly plants. An additional ecological role has been proposed for *Mortierella* spp. because of the high activities of chitinases and serine proteases of the cold-tolerant *Mortierella* species, indicating an insecticidal potential [24]. Our results showed that *M. antarctica* and *M. fimbricystis* were efficiently growing on casein and insoluble phosphate-containing medium (Supplementary Figure S1).

Besides *Mortierella* spp., the fungal communities isolated from Antarctic lakes contained other endemic as well as cold-adapted cosmopolitan genera such as *Mrakia* and *Penicillium* [18,19]. In this study, some fungi species from these genera were found exclusively in *D. antarctica* roots (*Penicillium melanoconidium*) and some in both grasses (*Mrakia frigida*). *Mrakia* has been found in many cold environments throughout the world; notably, *Mrakia* is the dominant yeast genus in Antarctic soil [25]. The optimal growth temperature for *Mrakia* spp. is approximately 12–15 °C, and they produce cold-active enzymes responsible for the involvement in nutrient cycling in glacier habitats [25]. 

In *P. annua* roots, we found another Ascomycete yeast, *Vishniacozyma victoriae*, previously isolated from Antarctic lichens [26]. Lichens are mainly associated with rocky substrates that have to be solubilized to mobilize the biogenic elements. *V. victoriae* was able to release tartaric and acetic acids and, similarly to *Mortierella* spp., revealed high phosphate solubilization activity. This yeast genus was also found as endophytes of some fruits, conferring resistance to the pathogenic fungus *Botrytis cinerea* [27]. In this study, this pathogen was found in the soil of the Ecology Glacier foreland (Table 1). 

Besides the *Vishniacozyma* and *Mrakia* species, several other yeast species were identified in the grasses studied here. It has been shown that *Leucosporidium creatinivorum* (roots of *P. annua*) and *L. fragarium* (roots of both grasses) are characterized by a high content of polyunsaturated fatty acids, which is a typical adaptation to a cold environment [32]. Recently, *L. creatinivorum* was analyzed with other Antarctic yeasts (including *V. victoriae*) for the production of antifreeze proteins, fatty acids, and ergosterol [32]. *L. creatinovorum* and *V. victoriae* contained high amounts of ergosterol compared with the other yeast species. Interestingly, no significant changes in total sterol content were observed when this yeast was grown at different temperatures [32].

Some of the grass-associated fungi identified in the presented study, including *Volucrispora graminea* (*Ypsilina graminea*), and *Oculimacula yallundae* (roots from both grasses), have been described as cereal pathogens [33,34] or, in the case of *Fusicladium* genus (roots from both grasses), as plant pathogens infecting fruit trees (pear, peach, almond, pecan, apple) [35]. *Fusicladium* sp. has not been reported from Antarctica to date. 

*Microdochium fragmitis*, the airborne fungus found in *P. annua* roots, was recognized as a *Fusarium*-like fungus that can be a pathogen of grasses, including cereals [38]. 

Some highly specialized fungi were recruited only by *D. antarctica*, e.g., the black yeast *Cladophialophora minutissima*, one of the most tolerant multiextremophiles on the planet [39]. It is able to adapt to and reproduce in the harshest conditions such as extreme dryness, high UV and ionizing radiation, oil contamination, high and low temperatures, and oligotrophy. It was previously found in Antarctic soil [40]. It is perfectly adapted to life on and within rocks [41]. 

Another fungus found only in *D. antarctica*, *Clitopilus* sp., has been reported to establish an ectomycorrhizal association with plants in the presence of organic nitrogen, as was shown for *C. hobsonii* and its host *Populus tomentosa* [42]. 

In conclusion, root-associated mycobiomes differed between the Antarctic-native *D*. *antarctica* and the Antarctic-invasive *P*. *annua* in phylogenetic diversity, community structure, and responses to changing edaphic conditions. *D*. *antarctica*, while not having an indispensable, fixed set of beneficial fungal taxa, tolerated a biodiverse microbiome, likely containing species with beneficial and pathogenic traits alike. Because of its long history of interaction with Antarctic soil microbiota, it was able to recruit biocontrol agents to promote inter-species competition, thus shifting the structure of its resident community towards the dominance of growth-promoting or at least benign endophytes. On the other hand, in *P*. *annua* roots, a core mycobiome was present that always substantially contributed to the endophyte community. Although its role is not clear, based on available data, we can assume that it alleviates cold-induced stress by modulating innate host plant responses, thus increasing the competitiveness of *P*. *annua* in Antarctic settings. Both grass species housed a plethora of fungal strains with different plant-beneficial traits, most notably, those with strong chitinolytic abilities that can be crucial in controlling phytopathogenic fungi, nematodes, and insects. Cold-adapted enzymes secreted by those endophytes could be of value in biotechnology, especially in light of greenhouse gas emission reduction. 

## 4. Material and Methods

### 4.1. Sites and Sampling

Samples were collected during the austral summer season of 2017–2018 from three sites on King George Island, South Shetland Islands, and maritime Antarctica (Table 2). Roots of *D. antarctica* and *P. annua* and their surrounding soil were collected from two locations within the Arctowski Station area and from the Ecology Glacier foreland. Two locations within Arctowski Station were chosen because they clearly differed in the presence of animals and, therefore, in the availability of nutrients for plants. Four to six specimens of the particular grass species were collected per site with the adjacent soil with the use of sterile tools, which were placed into sterile plastic containers and transported frozen (−20 °C) to the Institute of Biochemistry and Biophysics, Polish Academy of Sciences (Warsaw, Poland).

### 4.2. DNA Extraction, ITS1 Amplicon Library Preparation, and Sequencing

Three grass specimens of a particular species were analyzed per site. Three root samples per specimen were subjected to DNA extraction. For surface sterilization, the roots were immersed in 70% ethanol for 2 min followed by 5 min. in 5% sodium hypochlorite, and then rinsed three times (1 min. each) with sterile distilled water [42]. After drying on sterile filter paper, the roots were cut, and 100 mg samples were homogenized in liquid nitrogen and used for DNA isolation using Plant DNA Mini Kit (Syngen Biotech, Wrocław, Poland), following the manufacturer’s protocol. The quality of the DNA was checked in 1% TAE-agarose gel stained with ethidium bromide. The resulting root DNA sample solutions (54 in total) were stored at −20 °C until further processing. The roots were also collected for the cultivation of fungi for further analysis.

Six soil samples per site were processed (three samples from the vicinity of each grass species occurrence). Rhizosphere soil DNA was extracted using the PowerSoil^®^ DNA isolation kit (QIAGEN GmbH, Hilden, Germany) according to the manufacturer’s protocol. Approx. 0.2 g of soil was used in triplicates per sample. DNA was kept in solution at −20 °C for further analysis. To guarantee the best DNA quality, different kits were used to isolate DNA from plants and soil, dedicated specifically to this type of sample.

Because of the low yield of the obtained DNA, the samples were pooled and concentrated, resulting in 18 root DNA samples (3 specimens × 2 species × 3 sites) and 6 soil DNA samples (2 species × 3 sites). Those samples were subjected to PCR amplification and high-throughput sequencing. Fungal taxa were identified based on the Internal Transcribed Spacer 1 sequence (ITS1) within the nuclear rDNA region [61,62]. The ITS1 region was amplified in total volume of 25 µL using 5 µL of 1 µM primers ITS1FI2 and 5.8S (Table 3) [63,64]. PCR reactions were performed using 2.5 µL (5 ng/µL) of DNA template, 12.5 µL of 2xKAPA HiFi Hot Start Ready Mix (Kapa Biosystems, Wilmington, MA, USA), and the following conditions: 95 °C for 3 min, followed by 25 cycles of 95 °C for 30 s, 55 °C for 30 s, 72 °C for 30 s, with a final extension step at 72 °C for 5 min. Sequencing of the PCR products was performed by Genomed (Warsaw, Poland) using an Illumina MiSeq Instrument (Analityk, Warsaw, Poland) and paired-end (2 × 250 bp) mode with the V2 Illumina kit (Analityk, Warsaw, Poland) [65]. A control reaction was performed without added DNA. 

### 4.3. Cultivation of Fungi from Roots and Soil and DNA Extraction 

To culture root-inhabiting fungi, root samples collected as described above were transferred to Petri dishes containing PDA medium (Potato Dextrose Agar) supplemented with chloramphenicol (0.01%), to inhibit bacterial growth, and Bengal Rose (0.005%), to prevent overgrowth by rapidly growing molds and to facilitate isolation of slow-growing fungi [67], followed by cultivation at 10 °C. Single fungal colonies were sub-cultured on separate Petri dishes containing PDA medium to obtain pure isolates for further studies. Pure culture isolates were deposited at the culture collection of the Laboratory of Fungal Biology IBB PAS, Warsaw, Poland. 

To cultivate fungi from rhizosphere soil, a 2 g soil sample was taken to the 50 mL Falcon tube and 4 mL of sterile water was added. After vigorous vortexing, a portion of the liquid was transferred to a Petri dish containing PDA, chloramphenicol, and Bengal Rose and cultivated as above. 

For DNA extraction, mycelia were cultivated in PDB medium (Potato Dextrose Broth) at 10 °C on a rotary shaker (250 rpm) in 250 mL shake flasks containing 100 mL of medium. DNA was isolated using the Wizard Genomic DNA Purification kit (Promega, Mannheim, Germany). Extracted DNA was used as the template for the amplification of the ITS region using universal primers ITS1 and ITS4 and a standard PCR protocol [64,66]. Amplified products were separated by electrophoresis on an agarose gel, isolated from the gel by Qiaex II Gel extraction kit (Qiagen), and sequenced. DNA sequences were analyzed using the NCBI database and the BLAST algorithm [68].

Identified species were classified according to the MycoBank fungal database (nomenclature and species bank) http://www.mycobank.org (accessed on 11 May 2024). Sequences were deposited in the GenBank database under the accession numbers PP761231-PP761248.

### 4.4. Screening for Phosphate Solubilization Ability

The ability of fungi to perform phosphate solubilization was assayed on a solid medium pH = 7 containing 1 L: 10 g glucose; 5 g MgCl_2_ × 6H_2_O; 0.25 g MgSO_4_ × 7H_2_O: 0.2 g KCl; 0.1 g (NH_4_)_2_SO_4_; and 5 g of Ca_3_(PO_4_)_2_ as the sole phosphate source. Only fungi capable of releasing soluble phosphate from insoluble tricalcium phosphate grew on such a medium. The formation of a halo indicative of the activity in question was evaluated after four weeks of incubation at 10 °C [26]. The screening was carried out in triplicate. 

### 4.5. Evaluation of Proteolytic Activity of Fungi 

Proteolytic activity was analyzed by growing fungi on a solid medium containing 1% skimmed milk in 50 mM Tris-HCl buffer and 1% agar. A 0.5 cm agar disc containing fungal culture was put in the center of the plate and incubated at 10 °C for 4 weeks. Proteolytic activity was indicated by the formation of a clear halo around the colony [69]. The diameter of the fungal colony and the colony plus halo was measured every week. 

### 4.6. Screening for Chitinase, Xylanase, Amylase, Cellulase, and Pectinase Activity 

Cultivable fungal strains were analyzed for their ability to produce specific extracellular hydrolases by cultivation on minimal medium containing 1 L: 1 g MgSO_4_ × 7H_2_O, 6 g (NH_4_)_2_SO_4_, 10 g KH_2_PO_4_, 3 g sodium citrate × 2H_2_O, and trace elements (25 mg FeSO_4_ × 7H_2_O, 2.7 mg MnCl_2_ × 4H_2_O, 6.2 mg ZnSO_4_ × 7H_2_O, 14 mg CaCl_2_ × 2H_2_O) per liter and 20 g agar and 1 g chitin or 5 g xylan or 10 g starch or 10 g carboxymetylcellulose or 5 g pectin as a carbon source [70,71,72,73]. A five-millimeter agar disc containing fungal culture was put in the center of the plate and incubated at 10 °C for 4 weeks. The activities were detected as a clear zone around the colony. The clear zones indicating cellulolytic, chitinolytic, or xylanolytic activity were visualized by developing the plate with 0.1% Congo Red. The clear zones indicating amylolytic or pectinolytic activity were visualized by developing the plates with Lugol’s iodine solution.

### 4.7. Data Curation and Analysis 

Illumina sequence reads were initially quality-checked using the FastQC toolkit [74] and further processed using the Qiime2 pipeline [75]. In brief, paired reads were merged and denoised using DADA2, and Amplicon Sequence Variants (ASVs) were constructed. Sequences were then taxonomically classified using a Bayesian Naïve classifier pre-trained on the Unite v.9.0 database (https://unite.ut.ee/ (accessed on 1 July 2024)). Non-target (non-fungal) ASVs were filtered out. The core microbiome was defined as a set of specific fungal ASVs that occurred in every sample within a particular group (soil, *D. antarctica* roots, *P. annua* roots). Downstream analysis was performed in the R environment. Raw Illumina reads were deposited in the NCBI Sequence Read Archive (SRA) as BioProject PRJNA1122116. All results were compiled using Excel 2016 (MS Office) for Windows. The unpaired two-sample Wilcoxon test (also known as the Wilcoxon rank sum test or Mann–Whitney test) was used in all tests where significance (*p* < 0.05) was presented. This test is a non-parametric alternative to the unpaired two-sample t-test, which can be used to compare two independent groups of samples. It is used when data are not normally distributed. *p* values were adjusted using the Bonferroni correction method. Principal Component Analysis (PCA) of fungal communities based on ASV abundances was performed using the singular value decomposition method. Data visualization and statistical analysis were performed using R software (R v.4.0.2) and the following packages: ggplot2, pheatmap, ggvenn, fmsb, Hmisc, ggpubr, corrplot, and autoplot (R Core Team 2002) [76].

## 5. Conclusions

In this study, we compared the fungal communities inhabiting the roots of two Antarctic grasses, *D. antarctica*—a native to Antarctic—and the alien grass—*P. annua*. Root-associated mycobiomes differed between the Antarctic-native *D*. *antarctica* and the Antarctic-invasive *P*. *annua* in phylogenetic diversity, community structure, and responses to changing edaphic conditions. *D. antarctica* recruited more fungi compared with *P. annua*. ASV numbers in *D. antarctica* roots were significantly higher than those found in *P. annua* roots, and the fluctuations between samples were much lower for the samples from *D. antarctica*. Overall, *D. antarctica*, which has a long history in Antarctica, appears to have developed associations with a wide variety of fungi, and its root microbiome is more stable compared with *P. annua*. *D*. *antarctica*, while not having an indispensable, fixed set of beneficial fungal taxa, tolerated a biodiverse microbiome, likely containing species with beneficial and pathogenic traits alike. It was able to recruit biocontrol agents to promote inter-species competition, thus shifting the structure of its resident community towards the dominance of growth-promoting or at least benign endophytes. On the other hand, in *P*. *annua* roots, a core mycobiome was present that always substantially contributed to the endophyte community. Although its role is not clear, based on available data, we can assume that it alleviates cold-induced stress by modulating innate host plant responses, thus increasing the competitiveness of *P*. *annua* in Antarctic settings. Both grass species housed a plethora of fungal strains with different plant-beneficial traits, most notably, those with strong chitinolytic abilities, which can be crucial in controlling phytopathogenic fungi, nematodes, and insects. Cold-adapted enzymes secreted by those endophytes could be of value in biotechnology, especially in light of greenhouse gas emission reduction.

## Figures and Tables

**Table 1:** Cultivable fungi from Antarctic grasses and soil.

Fungal Species	Phylum	Characteristics	Reference
*D. antarctica* roots			
*Helgardia anguioides*-like (*Oculimacula*)	Ascomycota	causes eyespot disease of wheat	[16,17]
*Herpotrichia juniperi*	Ascomycota	causal agent of brown felt blight of conifers	[18]
*Sporobolomyces roseus*	Basidiomycota	synthesizes carotenoids and mycosporines to absorb UV radiation; secretes antimicrobial protein	[19]
*Ophiosphaerella* sp.	Ascomycota	grass pathogen	[20]
*P. annua* roots			
*Mortierella antarctica*-like	Mortierellomycota	promotes winter wheat seedling growth	[21]
*Microdochium phragmitis*	Ascomycota	endophyte colonizing roots of common reed	[22]
*Ophiosphaerella* sp.	Ascomycota	causes spring dead spot of bermudagrass	[23]
Soil—Ecology Glacier foreland			
*Cladosporium herbarum*	Ascomycota	source of antimicrobial compounds; effective in bioremediation of perfluorochemicals	[24,25]
*Botrytis cinerea*	Ascomycota	cosmopolitan broad host-range plant pathogen	[26]
*Mrakia robertii*	Basidiomycota	producer of cold-tolerant enzymes	[27]
*Leucosporidium* sp. *Camptobasidium gelus*	Basidiomycota	*Leucosporidium* sp.—yeast from Antarctic marine sponge, can biocontrol *B. cinerea* infection *Camptobasidium gelus*-yeast found in Svalbard and Greenland glacial	[28,29,30]
*Leucosporidiales* sp.	Basidiomycota		
Soil—Arctowski Station 2			
*Mortierella fimbricystis*	Mortierellomycota	tolerates low pH, low temperature, high concentration of metals, and low organic matter	[31]
*Oidiodendron truncatum*	Ascomycota	producer of antifungal metabolites	[32,33]
*Pseudoeurotium* sp.	Ascomycota	candidate for bioremediation of polluted soil	[24]
*Pseudoeurotium* sp.	Ascomycota	candidate for bioremediation of polluted soil	[24]
*Linnemannia hialina* (*Mortierella hyalina*)	Mortierellomycota	identified in iron ore mine as heavy metal-tolerating	[34]
*Cladosporium tenuissimum*	Ascomycota	candidate for bioremediation of polluted soil	[24]

**Table 2:** Main characteristics of the sampling sites. S1–S3—soil samples; D1–D3—*D. antarctica* root samples; P1–P3—*P. annua* root samples.

Site	Geographical Coordinates	Distance to the Sea	Sample ID	Landform and Habitat
Arctowski Station 1	62°09′35″ S 58°28′26″ W	120 m	S1, D1, P1	Scientific station area; soil mechanically altered by human activities; Skeletic Eutric Fluvisol (Turbic). The site is strongly influenced by marine aerosols; it is moist and located further from sources of nutrients, with large human influence.
Arctowski Station 2	62°09′33″ S 58°28′25″ W	100 m	S2, D2, P2	Scientific station area; soil mechanically altered by human activities; Skeletic Eutric Fluvisol (Turbic). The site is strongly influenced by marine aerosols; it is moist and close to animal breeding colonies, with large human influence, especially related to using heavy vehicles.
Ecology Glacier foreland	62°10′05″ S 58°27′46″ W	20 m	S3, D3, P3	Fluted moraine; Eutric Skeletic Protic Regosol (Turbic). Dry site, sheltered from sources of nutrients, with little influence of marine aerosols.

**Table 3:** Primers used for the amplification of rDNA regions.

Primer Name	5′-Sequence-3′	Purpose	Reference
ITS1FI2	GAACCWGCGGARGGATCA	Fungal-specific ITS1 amplification in the metagenomic study	[63]
5.8S	CGCTGCGTTCTTCATCG		[64]
ITS1	TCCGTAGGTGAACCTGCGG	Identification of cultured fungi	[66]
ITS4	TCCTCCGCTTATTGATATGC		

## Data Availability

Data is contained within the article and supplementary material.

## Supplementary Materials

The supporting information can be downloaded at: https://www.mdpi.com/article/10.3390/ijms25158475/s1.
