# Supplementary material for: Native and Alien Antarctic Grasses as a Habitat for Fungi
Source: Int J Mol Sci. 2024 Aug 3;25(15):8475. doi: 10.3390/ijms25158475 (PMC11313430; doi:10.3390/ijms25158475)
Supplement: Supplementary file 1 [file ijms-25-08475-s001.zip › ijms-3128117-supplementary.pdf]

A

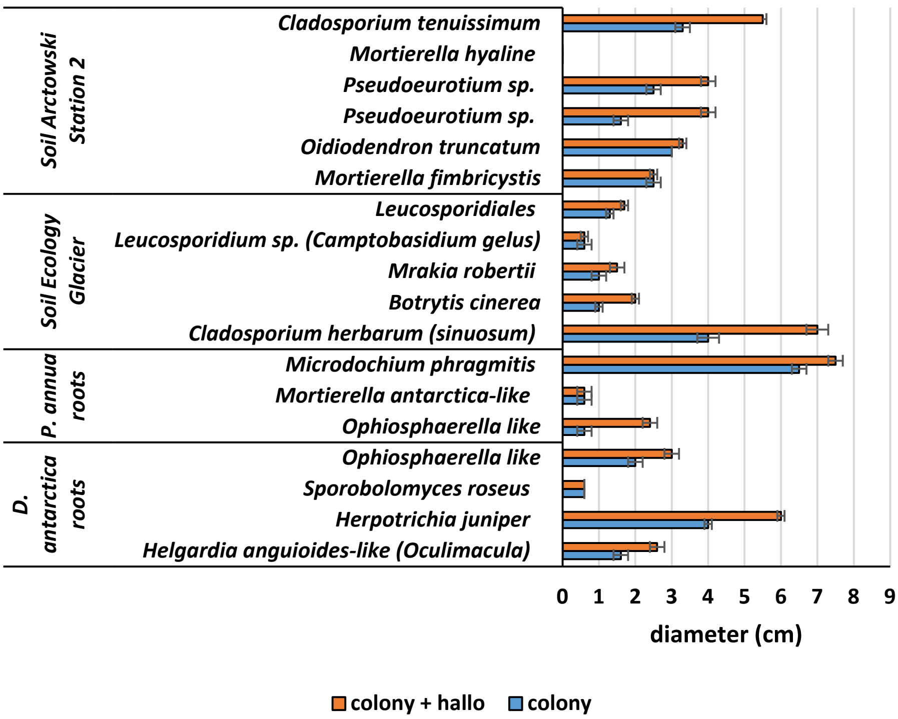
Soil Arctowski Station 2
Cladosporium tenuissimum
Mortierella hyaline
Pseudoeurotium sp.
Pseudoeurotium sp.
Oidiodendron truncatum
Mortierella fimbricystis
Soil Ecology Glacier
Leucosporidiales
Leucosporidium sp. (Camptobasidium gelus)
Mrakia robertii
Botrytis cinerea
Cladosporium herbarum (sinuosum)
P. annua roots
Microdochium phragmitis
Mortierella antarctica-like
Ophiosphaerella like
D. antarctica roots
Ophiosphaerella like
Sporobolomyces roseus
Herpotrichia juniper
Helgardia anguioides-like (Oculimacula)
0 1 2 3 4 5 6 7 8 9
diameter (cm)
colony + hallo
colony

B

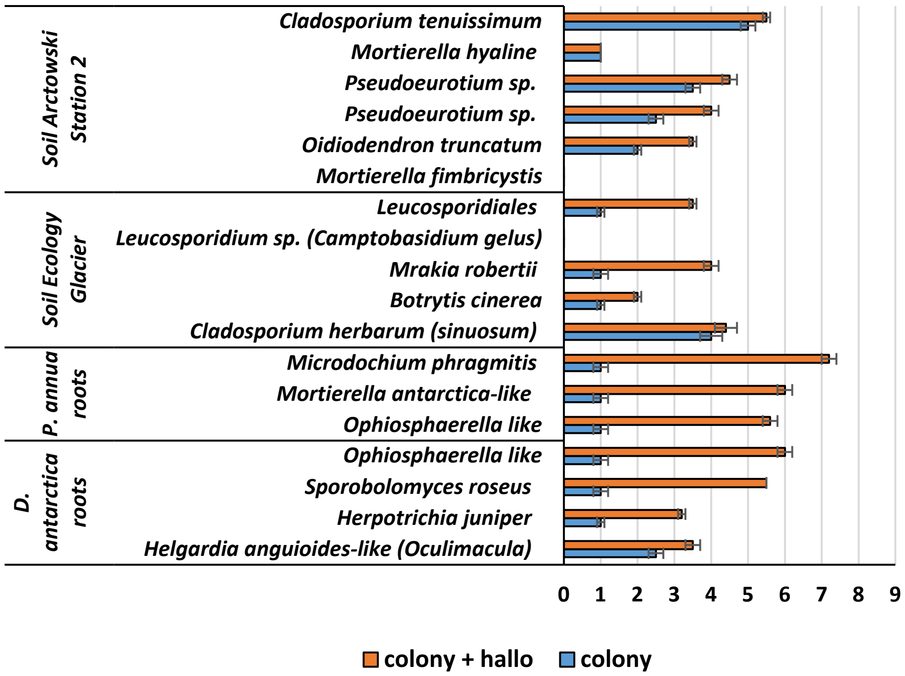
Soil Arctowski Station 2
Cladosporium tenuissimum
Mortierella hyaline
Pseudoeurotium sp.
Pseudoeurotium sp.
Oidiodendron truncatum
Mortierella fimbricystis
Soil Ecology Glacier
Leucosporidiales
Leucosporidium sp. (Camptobasidium gelus)
Mrakia robertii
Botrytis cinerea
Cladosporium herbarum (sinuosum)
P. annua roots
Microdochium phragmitis
Mortierella antarctica-like
Ophiosphaerella like
D. antarctica roots
Ophiosphaerella like
Sporobolomyces roseus
Herpotrichia juniper
Helgardia anguioides-like (Oculimacula)
0 1 2 3 4 5 6 7 8 9
colony + hallo
colony

C

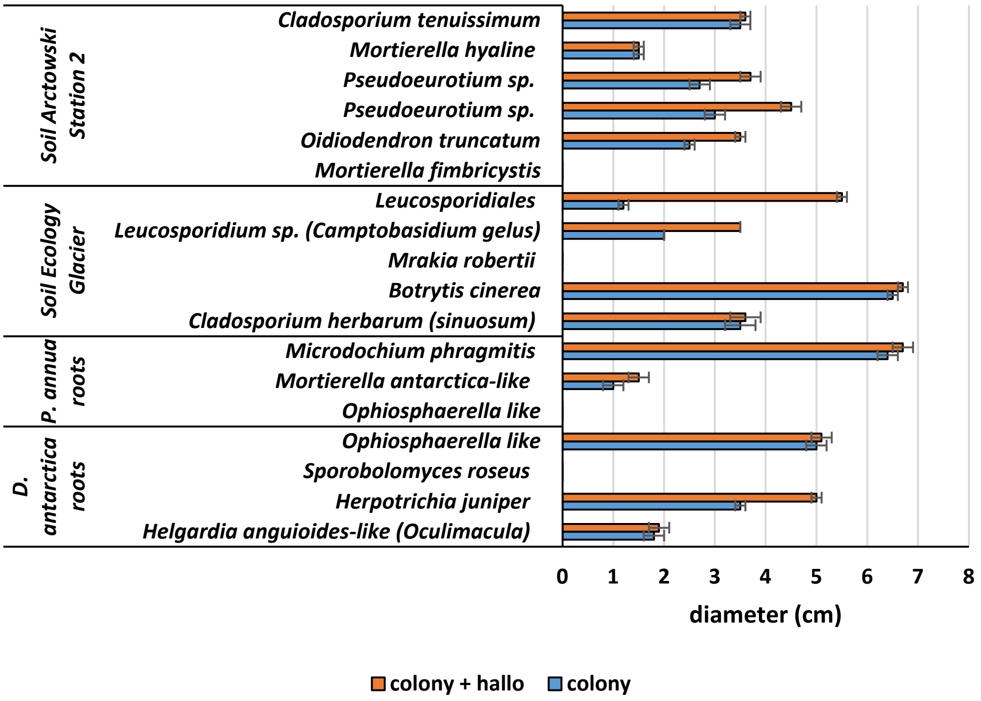
Soil Arctowski Station 2
Cladosporium tenuissimum
Mortierella hyaline
Pseudoeurotium sp.
Pseudoeurotium sp.
Oidiodendron truncatum
Mortierella fimbricystis
Soil Ecology Glacier
Leucosporidiales
Leucosporidium sp. (Camptobasidium gelus)
Mrakia robertii
Botrytis cinerea
Cladosporium herbarum (sinuosum)
P. annua roots
Microdochium phragmitis
Mortierella antarctica-like
Ophiosphaerella like
D. antarctica roots
Ophiosphaerella like
Sporobolomyces roseus
Herpotrichia juniper
Helgardia anguioides-like (Oculimacula)
0 1 2 3 4 5 6 7 8
diameter (cm)
colony + hallo
colony

D

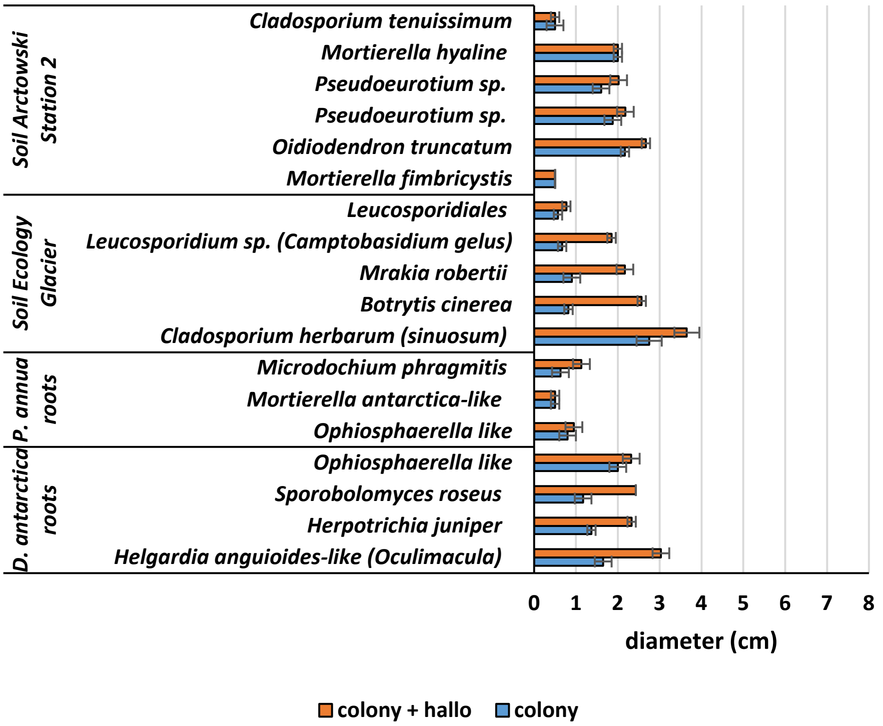
Soil Arctowski Station 2
Cladosporium tenuissimum
Mortierella hyaline
Pseudoeurotium sp.
Pseudoeurotium sp.
Oidiodendron truncatum
Mortierella fimbricystis
Soil Ecology Glacier
Leucosporidiales
Leucosporidium sp. (Camptobasidium gelus)
Mrakia robertii
Botrytis cinerea
Cladosporium herbarum (sinuosum)
P. annua roots
Microdochium phragmitis
Mortierella antarctica-like
Ophiosphaerella like
D. antarctica roots
Ophiosphaerella like
Sporobolomyces roseus
Herpotrichia juniper
Helgardia anguioides-like (Oculimacula)
0 1 2 3 4 5 6 7 8
diameter (cm)
colony + hallo
colony

E

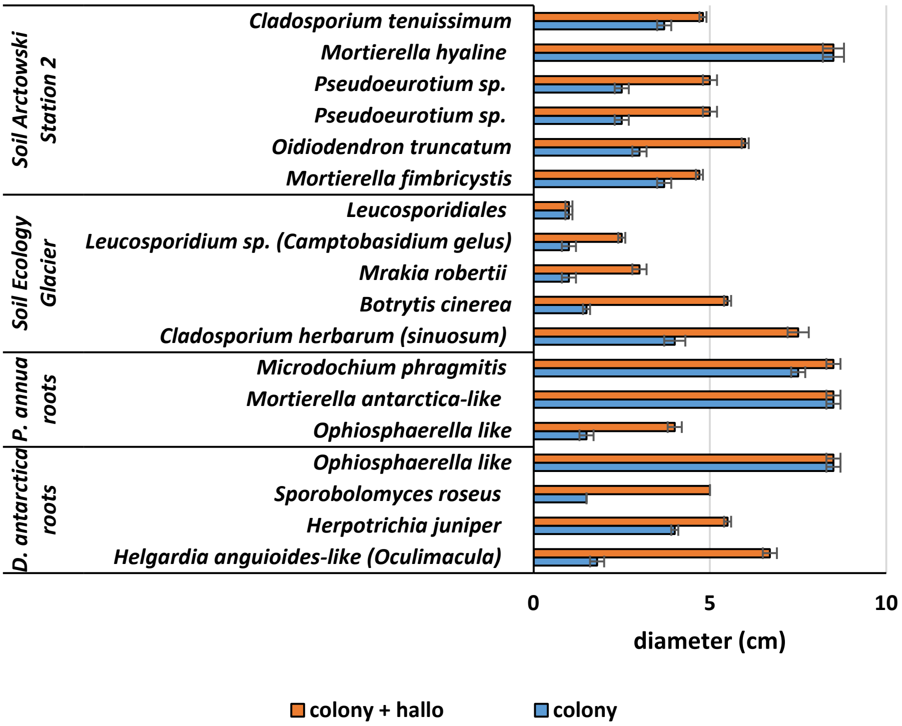

Figure S1
Hydrolytic activity of fungi cultivated at 10°C on Petri dishes containing minimal medium with carboxymethylcellulose (A), chitin (B), xylan (C), starch (D) or pectin (E) as a carbon source (Section 2.5). After 4 weeks of cultivation plates were developed over night with 0.1% Congo Red for chitinolytic, xylanolytic and cellulolytic activities to visualize clear zone of hydrolyzed substrates. The clear zones indicating amylolytic and pectinolytic activities were visualized by developing the plates with Lugol's iodine solution. Next, the diameter of the fungal colony and colony plus hallo was determined.

Table S1. ASV identified to the species level in *D. antarctica* and *P. annua* roots

	D. antarctica	*P. annua*	**Phylum**
1.	*Cladophialophora_minutissima*		Ascomycota
2.	*Volucrispora_graminea*	*Volucrispora_graminea*	Ascomycota
3.	*Malassezia_restricta*	*Malassezia_restricta*	Ascomycota
4.	*Pseudogymnoascus_destructans*	*Pseudogymnoascus_destructans*	Ascomycota
5.	*Pseudogymnoascus_roseus*		Ascomycota
6.	*Trichoderma_citrinoviride*		Ascomycota
7.	*Verrucaria_humida*	*Verrucaria_humida*	Ascomycota
8.	*Verrucaria_alpicola*	*Verrucaria_alpicola*	Ascomycota

9. Fulvoflamma_eucalypti		Ascomycota
10. Lachnum_carneolum		Ascomycota
11. Ascomycotacoryne_sarcoides		Ascomycota
12.	*Vishniacozyma_victoriae*	Basidiomycota
13.	*Mrakia_niccombsii*	Basidiomycota
14. Xenopolyscytalum_pinea		Ascomycota
15.	*Myrmecridium_hiemale*	Ascomycota
16. Leucosporidium_fragarium	*Leucosporidium_fragarium*	Basidiomycota
17.	*Leucosporidium_creatinivorum*	Basidiomycota
18. Placynthiella_oligotropha		Ascomycota
19. Lecania_glauca		Ascomycota
20. Buellia_russa	*Buellia_russa*	Ascomycota
21. Clitopilus_hobsonii		Basidiomycota
22. Lachnellula_fuscosanguinea	*Lachnellula_fuscosanguinea*	Ascomycota
23. Trichoderma_virilente		Ascomycota
24.	*Naganishia_vaughanmartiniae*	Basidiomycota
25.	*Naganishia_albida*	Basidiomycota
26. Mrakia_frigida	*Mrakia_frigida*	Basidiomycota
27. Alternaria_metachromatica		Ascomycota
28.	*Alternaria_angustiovoidea*	Ascomycota
29. Fusicladium_fagi	*Fusicladium_fagi*	Ascomycota
30. Lecanora_polytropa		Ascomycota
31. Rachicladosporium_mcmurdoi	*Rachicladosporium_mcmurdoi*	Ascomycota
32. Cosmospora_arxii		Ascomycota
33. Tulostoma_striatum		Basidiomycota
34. Mortierella_elongatula	*Mortierella_elongatula*	Morterellomycota
35.	*Mortierella_gamsii*	Morterellomycota
36. Mortierella_amoeboidea		Morterellomycota
37. Mortierella_antarctica	*Mortierella_antarctica*	Morterellomycota
38.	*Mortierella_angusta*	Morterellomycota
39.	*Mortierella_hyalina*	Morterellomycota
40. Mortierella_jenkinii		Morterellomycota
41. Mortierella_minutissima		Morterellomycota
42. Mortierella_basiparvispora		Morterellomycota
43. Phenoliferia_psychrophila	*Phenoliferia_psychrophila*	Basidiomycota
44. Neodevriesia_capensis	*Neodevriesia_capensis*	Ascomycota
45. Sistotrema_autumnale		Basidiomycota
46. Itersonilia_perplexans		Basidiomycota
47.	*Calycina_alstrupii*	Ascomycota
48. Rinodina_olivaceobrunnea	*Rinodina_olivaceobrunnea*	Ascomycota
49. Psathyrella_echinata		Basidiomycota
50. Hyaloscypha_bicolor		Ascomycota
51. Cordyceps_bassiana		Ascomycota
52. Ophiocordyceps_robertsii		Ascomycota
53. Bacidina_sulphurella		Ascomycota
54. Oidiodendron_chlamydosporicum		Ascomycota

55. Oidiodendron_truncatum		Ascomycota
56.	Dactylaria_dimorphospora	Ascomycota
57. Agonimia_repleta		Ascomycota
58. Solicoccozyma_terricola		Basidiomycota
59. Micarea_denigrata		Ascomycota
60. Placynthiella_uliginosa		Ascomycota
61. Acremonium_biseptum	Acremonium_biseptum	Ascomycota
62. Acremonium_persicinum		Ascomycota
63. Trichocladium_opacum		Ascomycota
64. Aspergillus_subversicolor		Ascomycota
65. Leptosphaeria_veronicae	Leptosphaeria_veronicae	Ascomycota
66. Acarospora_austroshetlandica		Ascomycota
67. Penicillium_jamesonlandense		Ascomycota
68. Bannozyma_arctica		Basidiomycota
69. Adisciso_yakushimense		Ascomycota
70. Tausonia_pullulans	Tausonia_pullulans	Basidiomycota
71. Athelia_acrospora		Ascomycota
72. Cadophora_melinii	Cadophora_melinii	Ascomycota
73. Kondoa_changbaiensis		Basidiomycota
74. Ochrolechia_frigida	Ochrolechia_frigida	Ascomycota
75. Troposporella_monospora		Ascomycota
76.	Dactylellina_arcuata	Ascomycota
77.	Lambertella_tubulosa	Ascomycota
78. Exophiala_equina		Ascomycota
79.	Debaryomyces_prosopidis	Ascomycota
80. Sterigmatobotrys_macrocarpa		Ascomycota
81. Mycosphaerella_tassiana	Mycosphaerella_tassiana	Ascomycota
82. Psychronectria_hyperantarctica		Ascomycota
83. Cytosporella_juncicola	Cytosporella_juncicola	Ascomycota
84. Amylocorticiellum_molle	Amylocorticiellum_molle	Basidiomycota
85. Phialocephala_fortinii		Ascomycota
86. Solicoccozyma_terrea		Basidiomycota
87. Fusicolla_aquaeductuum		Ascomycota
88. Paraphoma_fimeti		Ascomycota
89. Exophiala_moniliae		Ascomycota
90. Blumeria_graminis		Ascomycota
91. Rickenella_fibula		Basidiomycota
92. Peziza_badia		Ascomycota
93. Omphalina_rivulicola		Basidiomycota
94. Phlebia_tremellosa		Basidiomycota
95. Xylodon_crustosus		Basidiomycota
96.	Gondwania_sejongensis	Ascomycota
97. Bryoglossum_gracile		Ascomycota
98. Boletus_luridus		Basidiomycota
99. Sistotrema_oblongisporum		Basidiomycota
100. Cadophora_luteo-olivacea		Ascomycota

101.Aspergillus_penicillioides		Ascomycota
102.Penicillium_melanoconidium		Ascomycota
103.Lactarius_glyciosmus		Basidiomycota
104.	*Oculimacula_yallundae*	Ascomycota
105.Hyphoderma_subsetigerum		Basidiomycota
106.Glaciozyma_martinii	*Glaciozyma_martinii*	Basidiomycota

Table S2. Cultivable fungi from Antarctic grasses and soil. Percentage of identity of the ITS region with sequences from the GenBank

**Fungal species**	**GeneBank accession No**	**ITS region identity[%] GenBank Acc. No**
***D. antarctica* roots**		
Helgardia anguioides-like (Oculimacula)	PP761231	Uncharacterized new species of *Plottnerulaceae*; 96% *Helgardiomyces anguioides* (MH861290)
Herpotrichia juniperi	PP761232	Uncharacterized new species of *Melanommataceae*; 96% *H. juniperi* (FJ904486)
Sporobolomyces roseus	PP761233	100% KU504594
Ophiosphaerella sp.	PP761234	99% KT269297
***P. annua* roots**		
Mortierella antarctica	PP761235	99% LC515021
Microdochium phragmitis	PP761236	99% MN077456
Ophiosphaerella sp.	PP761237	99% KT269297
**Soil –Ecology Glacier foreland**		
Cladosporium sp.	PP761238	*C. herbarum* 99% ON712161, *C. sinuosum* 100% ON712549
Botrytis cinerea	PP761239	100% MT150132
Mrakia robertii	PP761240	99% MT048630, 99% MT048621
Leucosporidiales sp. *Camptobasidium gelus*	PP761241	*Camptobasidium gelus* 100% NR_170718 Uncharacterized *Leucosporidiales* 99% FM997952
Leucosporidiales sp.	PP761242	Uncharacterized new species of *Leucosporidiales* 99% FM997952
**Soil – Arctowski station 2**		
Mortierella fimbricystis	PP761243	98% MH791155; 98% MT521789
Oidiodendron truncatum	PP761244	100% LC317709
Pseudoeurotium sp.	PP761245	Uncharacterized 100% MK543204
Pseudoeurotium sp.	PP761246	98% MK543204
Linnermannia hyalina, Mortierella hyalina	PP761247	99% KJ702042
Cladosporium tenuissimum	PP761248	99% KJ702042
